# Phase II proof-of-concept study of durvalumab and cediranib with and without olaparib in recurrent ovarian cancer

**DOI:** 10.21203/rs.3.rs-7724642/v1

**Published:** 2025-10-05

**Authors:** Jung-Min Lee, Junya Tabata, Tzu-Ting Huang, Elena Giudice, Kristen Ibanez, Jayakumar Nair, Aanika Warner, Britanny Solarz, Valentina Bolanos, Bernadette Redd, Nahoko Sato, Shraddha Rastogi, Sunmin Lee, Roshan Shrestha, Alexander Mitrophanov, Stanley Lipkowitz, Kevin Conlon, Chien Chu Huang

**Affiliations:** NCI; NCI; Women’s Malignancies Branch, Center for Cancer Research, National Cancer Institute, Bethesda, MD, USA; NCI; NCI; Women’s Malignancies Branch, Center for Cancer Research, National Cancer Institute, Bethesda, MD; NCI; NCI; NCI; Clinical Center, National Institutes of Health; Kumamoto University; National Cancer Institute; National Cancer Institute; National Institutes of Health; National Institutes of Health; National Cancer Institute; NCI; NCI

## Abstract

Platinum-resistant epithelial ovarian cancer (EOC) represents a population with limited therapeutic options. We conducted a proof-of-concept, phase II single-center, multi-arm study of durvalumab plus cediranib (D + C) or durvalumab, cediranib, and olaparib (D + O + C) in recurrent EOC. Sixty-eight patients were enrolled (D + O + C [n = 39] and D + C [n = 29]). Pre- and on-treatment biopsies and blood samples were collected for translational studies. Objective response rate was 19.4% (95% CI: 8.2–36.0) in D + O + C and 29.6% (95% CI: 13.8–50.2) in D + C. Progression-free survival (PFS) was 4.5 months for both arms. Four exceptional responders (PFS ≥ 12 months) were observed in each arm. Pre-treatment transcriptomic analysis identified that patients with exceptional response or clinical benefit (PR + SD ≥ 4 months) in both D + O + C and D + C arms demonstrated strong immune activation at baseline while D + C additionally depends on metabolic activity for response. Conversely, cytoskeletal redistribution was seen in transcriptomic data from patient tumors without clinical benefit. These findings emphasize the importance of combining immune, metabolic and cytoskeletal profiling-based treatment strategies for the future clinical studies in recurrent EOC.

## Introduction

Epithelial ovarian carcinoma (EOC) represents the most lethal gynecologic malignancy worldwide^1^. Nearly 75% of patients present with an advanced disease, and more than two third (~ 70%) experience disease recurrence within 3 years^2^. Standard of care therapy (SOC) for relapsed EOC involves platinum-based combination treatment (with paclitaxel, gemcitabine, or pegylated doxorubicin [PLD])^3, 4^ with or without bevacizumab for platinum-sensitive disease^5, 6^. In the platinum-resistant setting, bevacizumab in combination with paclitaxel, PLD, or topotecan has demonstrated improved progression-free survival (PFS) but a limited overall survival (OS) benefit^7^. Mirvetuximab soravtansine (MIRV), a folate receptor α (FRα) targeting antibody-drug conjugate (ADC), was the first ADC showing OS advantage compared to SOC in this setting, but its activity is restricted to tumors with high expression of FRα and high-grade serous histology^8^. As such, further options are needed for the remaining EOC, platinum-resistant population. Nab-paclitaxel plus relacorilant also has demonstrated PFS improvement, yet this treatment option was investigated only in a bevacizumab-pretreated platinum-resistant EOC population, excluding the primary platinum-refractory disease^9^. Collectively, these restrictions highlight the need for other therapeutic strategies for relapsed EOC.

Poly(ADP-ribose) polymerase inhibitors (PARPis) have been approved by the United States Food and Drug Administration (FDA) and the European Medicines Agency (EMA) as maintenance therapy for the newly diagnosed EOC responding to platinum-based chemotherapy^10, 11^. However, their use in later line settings has been restricted after the FDA revoked approvals beyond the third-line^12^, and the EMA declined approval, citing a lack of long-term survival benefit in the SOLO-3, ARIEL4, and QUADRA trials^13^. Although these studies reported longer PFS, the lack of OS improvement further demonstrates the difficulty of achieving durable responses in relapsed EOC, particularly with a monotherapy approach.

Immune checkpoint blockade such as the programmed death-ligand 1 (PD-L1)/PD-1 axis inhibitors has been investigated as a potential therapeutic strategy for EOC. However, immune checkpoint inhibitors (ICIs) have failed to show superiority compared to SOC treatments in EOC^14, 15^. This limited efficacy may be partly due to the immunosuppressive tumor milieu^16^, therefore non-chemotherapy-based ICI combination therapies may provide a novel angle to overcome challenges of ICI-based therapies^17^.

Preclinical data suggest PARPis may enhance tumor immunogenicity through increased tumor infiltration by cytotoxic T cells^18^, activation of the STING pathway^19^, and accumulation of unrepaired DNA double-strand breaks which promote recruitment of antigen-presenting cells^20^. Meanwhile, anti-angiogenic therapy has been shown to reduce immunosuppressive cell populations within the ovarian cancer microenvironment^21^. As such, concurrent PARP and anti-VEGF inhibition may boost the antitumor activity of ICIs by creating a favorable immune environment.

Early-phase clinical trials combining the VEGFR1–3 tyrosine kinase inhibitor (TKI) cediranib and the PARPi olaparib have demonstrated signal of activity in a subset of platinum-resistant EOC^22^. A subsequent phase I study incorporating durvalumab into olaparib and cediranib combination confirmed the tolerability of the triplet at the recommended phase II dose and showed preliminary clinical benefit in recurrent EOC^23^. However, the multi-center phase II trial (NRG-GY023; NCT04739800) failed to demonstrate improved clinical efficacy compared to SOC^24^. Notably, subsets of patients receiving ICI plus cediranib with or without olaparib exhibited exceptional responses (PFS ≥ 12 months) in this study, including three patients (6.8%) in the durvalumab, olaparib, and cediranib (D + O + C) arm and two (4.8%) in the durvalumab plus cediranib (D + C) arm. However, the trial lacked translational analyses to characterize those responders^24^.

In this report, we present the comprehensive translational research findings and clinical outcomes of recurrent EOC patients who received triple therapy with D + O + C, or doublet D + C from a single center, multi-arm, multi-cohort phase II study (NCT02484404). Moreover, we report mechanistic translational studies using transcriptome analysis on fresh biopsy samples and proof-of-concept preclinical studies. Our results suggest two distinct axes of clinical benefit in recurrent EOC such as an immune-primed axis for PARPi-containing immunotherapy and a complementary immunometabolic axis for anti-VEGF plus ICI combinations. Conversely, we find that therapeutic resistance converges on a shared biology of alternative vascularization and cytoskeletal plasticity. These findings emphasize the importance of integrating immune, metabolic, and cytoskeletal profiling to guide biomarker-driven treatment strategies for patients with recurrent EOC.

## Results

### Patient enrollment and baseline characteristics

**Figure 1:**
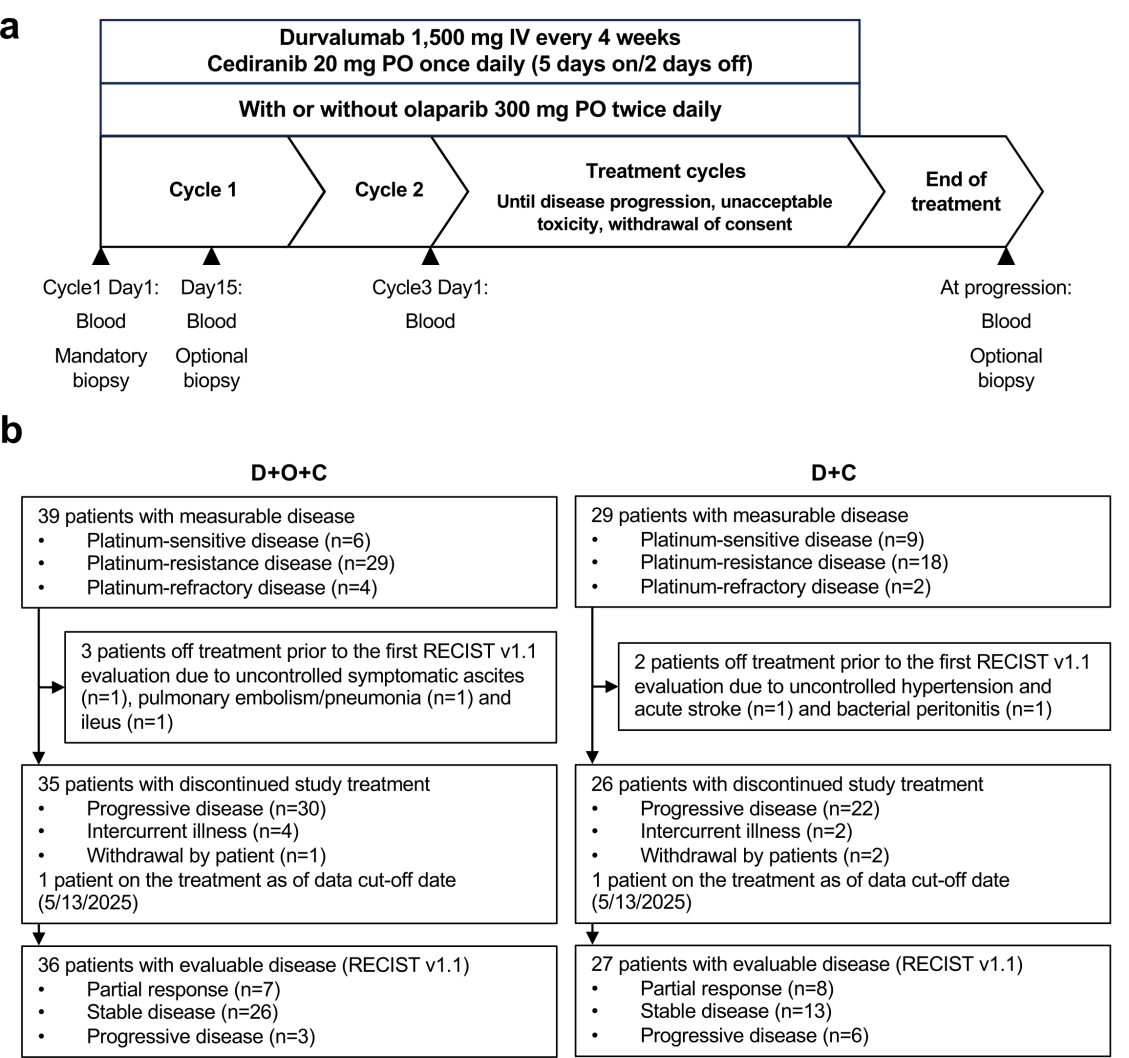
Clinical trial design with integrated exploratory analysis. **a.** Clinical study with correlative study endpoints: blood samples were collected prior to C1D1, C1D15, C3D1, and at progression. Tumor samples were collected prior to C1D1 (mandatory) and C1D15(optional) and at progression (optional). CT scans were performed at baseline and every 2 cycles (+/− 1 week) following treatment for RECIST evaluation. **b.** The CONSORT flow diagram. Overall, 39 patients in the D+O+C arm and 29 in the D+C arm were enrolled in the study. In the D+O+C arm, pre- and on-treatment biopsies were available in 34 and 11 patients, respectively. Three patients discontinued treatment prior to the first RECIST evaluation imaging due to intercurrent illness; (uncontrolled symptomatic ascites [n=1], pulmonary embolism/pneumonia [n=1] and ileus [n=1]. 36 patients were evaluated for tumor response per RECIST v1.1 criteria. In the D+C arm, pre- and on-treatment biopsy were available in 25 and 9 patients, respectively. Two patients discontinued treatment before the first RECIST evaluation imaging due to uncontrolled hypertension [n=1] and acute stroke and bacterial peritonitis [n=1]. 27 patients were evaluated for tumor response. Abbreviations: C1D1, cycle 1 day 1; C1D15, cycle 1 day 15; C3D1, cycle 3 day 1; D+O+C, durvalumab, cediranib, and olaparib; D+C, durvalumab plus cediranib; IV, intravenous; PO, orally.

Between September 2016 and August 2024, sixty-eight recurrent ovarian cancer patients were enrolled and received at least one dose of treatment (39 patients in the D + O + C arm and 29 in the D + C arm) (Fig. 1a–b). Baseline characteristics of each arm are detailed in Table 1. Briefly, D + O + C arm enrolled EOC histology (high-grade serous [87.2%] and clear cell [12.8%]). The majority of patients (~ 85%) had a *BRCA* wild-type and platinum-resistant or primary platinum-refractory disease. Also, a majority (~ 80%) had received prior bevacizumab. Of note, we previously reported the findings of the durvalumab plus olaparib (D + O) arm, which had similar baseline clinical characteristics, except for a lower rate of prior bevacizumab exposure (46%)^25^.

D + C arm also enrolled patients with mostly EOC histology (high-grade serous [72.4%], clear cell [6.9%], and high-grade endometrioid [3.4%]). 86.2% of patients were *BRCA* wild-type, and 69% of participants presented with platinum-resistant or primary platinum-refractory disease. About 59% of patients had received prior bevacizumab. The median number of prior treatments was three for both D + O + C and D + C arms, representing a heavily pretreated population.

### Efficacy and safety

**Figure 2:**
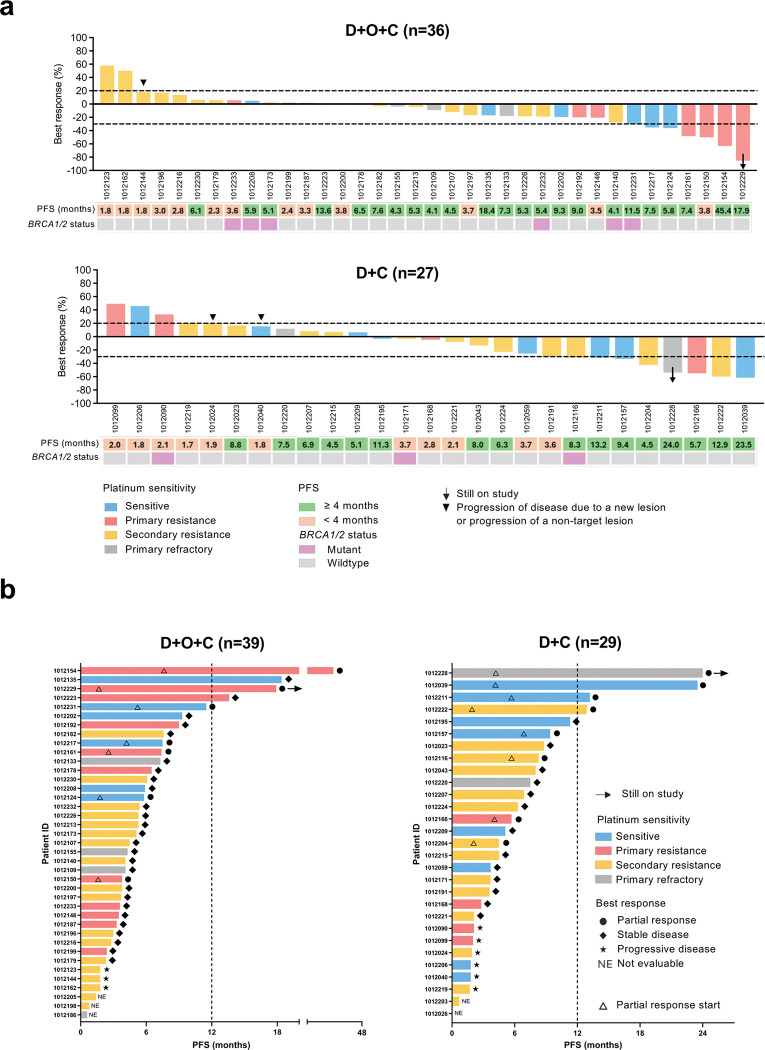
Antitumor activity. **a.** Waterfall plots of the best responses in RECIST-evaluable patients: The patients in the D+O+C (n = 36) and D+C (n = 27) arms are shown, respectively. The horizontal dotted line indicates the threshold for partial response (30% reduction in tumor size from baseline) and progressive disease (20% increase in tumor size from baseline). Arrows indicate cases still on study. Solid black downward triangles denote PD, defined as a new lesion or progression of a non-target lesion. For each patient, PFS and BRCA1/2 mutation status are indicated below their respective bar. **b.** Swimmer plot showing the PFS for each individual RECIST-evaluable patient. Bar color indicates platinum sensitivity for each case. Symbols at the end of the bars denote best response (RECIST v1.1). For cases with best response of PR, upward triangles indicate PR start. Abbreviations: CB, clinical benefit; D+O+C, durvalumab, cediranib, and olaparib; D+C, durvalumab plus cediranib; ITT, intention-to-treat; NCB, no clinical benefit; PFS, progression-free survival; PD, progression disease; PR, partial response; SD, stable disease; RECIST v1.1, Response Evaluation Criteria in Solid Tumors version 1.1.

For the D + O + C arm, seven of 36 (19.4%, 95% confidence interval [CI]: 8.2–36.0) Response Evaluation Criteria in Solid Tumors version 1.1 (RECIST v1.1)-evaluable patients achieved partial response (PR). 26 patients (72.2%) had stable disease (SD) (Fig. 1b). In the D + C arm, eight of 27 (29.6%, 95% CI: 13.8–50.2) RECIST-evaluable patients had PR and 13 (48.1%) had SD (Fig. 1b). The clinical benefit (CB, defined as the proportion of patients with PR or SD lasting longer than 4 months) rate was 66.7% (7 PRs and 17 SD ≥ 4 months; 95% CI: 49.0–81.4) in the D + O + C arm, and 59.3% (8 PRs and 8 SD ≥ 4 months; 16/27, 95% CI: 38.8–77.6) in the D + C arm, respectively (Fig. 2a). Waterfall plots summarizing the best tumor response in RECIST-evaluable patients are shown in Fig. 2a.

PRs were noted in both treatment arms irrespective of platinum sensitivity or prior bevacizumab exposure. Of the seven participants with PRs in the D + O + C arm, four had primary platinum-resistant disease and three were platinum-sensitive. All seven PRs occurred in bevacizumab-pretreated patients. In the D + C arm, four were platinum-resistant (primary platinum-resistant [n = 1] and secondary platinum-resistant [n = 3]), one was primary platinum-refractory, and three were platinum-sensitive. Two PRs were observed in bevacizumab-pretreated patients.

In the intention-to-treat (ITT) population, the median PFS was 4.5 months (CI: 3.8–6.1) for the D + O + C arm (n = 39), and 4.5 months (CI: 3.6–8.0) for the D + C arm (n = 29) (Fig. 2b). At the time of data cut-off (May 13, 2025), there were two patients with ongoing responses (17.9 + months [PR] in the D + O + C arm and 24.0 + months [PR] in the D + C arm). Notably, there were four exceptional responders (defined as those with PFS ≥ 12 months) in the D + O + C arm (45.4 months [PR], 18.4 months [SD], 17.9 months [PR], 13.6 months [SD]) and four in the D + C arm (24.0 months [PR], 23.5 months [PR], 13.2 months [SD], 12.9 months [PR]). PFS for each RECIST-evaluable patient is illustrated in Fig. 2b.

Treatment-related AEs (TRAEs) occurring in ≥ 10% of patients are presented in Supplementary Table 1. Consistent with prior reports^24^, the most common TRAEs observed in both treatment arms were hematologic and gastrointestinal. The most frequent all-grade TRAEs was anemia (79.5%, 31/39) for D + O + C arm and diarrhea (58.6%, 17/29) for D + C arm.

### Upregulation of immune and metabolic pathways in patients with clinical benefit

**Figure 3:**
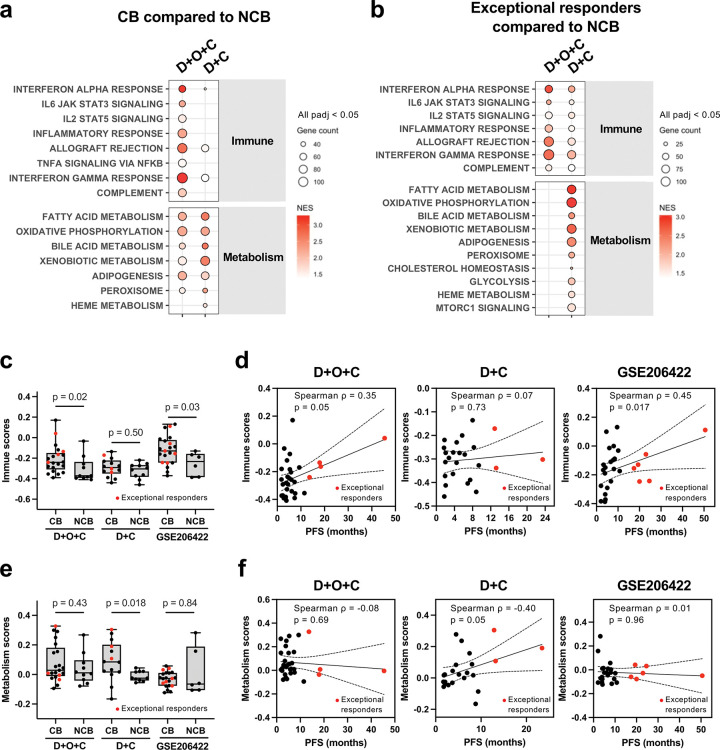
Baseline transcriptomic characteristics in patients with clinical benefit and exceptional response. **a-b**. Gene set enrichment analysis of bulk RNAseq data indicates pathways that may have contributed to clinical benefit (CB; PR or SD ≥ 4 months) (a) or exceptional response (PR or SD ≥ 12 months) (b) versus no clinical benefit (NCB; PD or SD < 4 months). Dot plot shows the enriched pathways associated with CB or exceptional response. Pathways with |NES| > 1.5, and padj < 0.05 are shown. Gene count represents the number of leading-edge genes contributing to each pathway. Circle size reflects gene count, with larger circles indicating more genes. Overlapping genes in selected pathways for NCB signature scoring are indicated by black outlined squares. **c-h.** Immune and metabolic signature scores in CB vs. NCB tumors within D+O+C (c-d), D+C (e-f), and a public dataset (GSE206422) in recurrent ovarian cancers with pembrolizumab, bevacizumab, and metronomic cyclophosphamide treatment (g-h) calculated using singscore and scores correlating with PFS. Two-sided Wilcoxon rank-sum test was used for comparing signature scores between CB and NCB. Spearman correlation test was used for correlation between signatures and PFS. Abbreviations: padj, adjusted p value; CB, clinical benefit; D+O+C, durvalumab, cediranib, and olaparib; D+C, durvalumab plus cediranib; NCB, no clinical benefit; NES, normalized enrichment score; PFS, progression-free survival; PD, progression disease; PR, partial response; SD, stable disease.

To identify transcriptomic characteristics of patient tumors with CB, we first analyzed RNA sequencing (RNAseq) data from pretreatment fresh biopsy samples. We performed Gene Set Enrichment Analysis (GSEA) Molecular Signature Database (MSigDB) hallmark and gene ontology biological function (GoBP) gene set collections^26^ to investigate pathways that may have contributed to treatment response. In both treatment arms, tumors from CB group (D + O + C [n = 21], D + C [n = 14]) exhibited significant upregulation of interferon alpha response (all adjusted p [padj] < 0.05; Fig. 3a and Supplementary Tables 2–3), suggesting a pre-existing immune-active microenvironment. The CB group from both treatment arms also showed enrichment of multiple metabolism-related pathways (all padj < 0.01, Fig. 3a), indicating a potential metabolic component of response. Of note, no individual gene was significantly associated with clinical outcome in either treatment arm based on the differential gene expression (DGE) analysis after multiple testing (padj = 0.77–1; Supplementary Tables 4–5), suggesting the value of pathway-level analyses over single-gene associations.

### Distinct immune and metabolic pathways dependencies in exceptional responders

While CB groups of both treatment arms showed upregulation of immune and metabolic pathways at baseline, we questioned whether this pattern was also found in tumors of exceptional responders. Compared to baseline transcriptomes of no clinical benefit (NCB) group (n = 10), D + O + C exceptional responders (n = 4) showed enrichment of immune pathways (all padj < 0.05) but without accompanying metabolic activation (Fig. 3b and Supplementary Table 6). In contrast, tumors of D + C exceptional responders (n = 3) had concurrent enrichment of immune (all padj < 0.05) and metabolic (all padj < 0.01) pathways relative to NCB tumors (n = 10; Fig. 3b and Supplementary Table 7).

Next, we used a single-sample ranking–based scoring method (singscore^27^) to quantify immune (Supplementary Tables 8–9) or metabolic activity (Supplementary Tables 10–11) in individual tumors. In the D + O + C arm, tumors from CB patients, including exceptional responders, had significantly higher immune scores than NCB tumors (Wilcoxon p = 0.02; Fig. 3c), and immune scores correlated positively with PFS (Spearman ρ = 0.35, p = 0.05; Fig. 3d). Exceptional responders consistently exhibited the higher immune scores within the CB group, suggesting a continuum of immune activity. Metabolic scores showed no association with benefit or PFS in D + O + C (Fig. 3e–f). In contrast, in the D + C arm, CB tumors had significantly higher metabolic scores than NCB tumors (Wilcoxon p = 0.018; Fig. 3e), and metabolic scores correlated with PFS (Spearman ρ = 0.40, p = 0.05; Fig. 3f). Exceptional responders again had the highest metabolic scores, reinforcing the link between metabolic activation and clinical benefit in this arm. Immune scores were not associated with benefit in D + C (Fig. 3c–d). PARP inhibition has been reported to induce DNA damage and cGAS-STING–dependent interferon signaling in *BRCA*-mutant breast and ovarian cancers^28, 29^, therefore we hypothesize that D + O + C treatment may require immunoreactive tumor milieu at baseline for its efficacy and less relying on a metabolically favorable baseline state, requiring further validation.

### Immune phenotype is also identified in an independent clinical trial dataset

To validate our immune signature, we studied the independent dataset from Rosario *et al.*^30^ which consisted of pretreatment RNAseq from a phase II trial of ICI (pembrolizumab), VEGF blockade (bevacizumab), and DNA damage response inhibitor (metronomic cyclophosphamide) combination therapy for ovarian cancer (GSE206422; NCT02853318). Using the same CB (n = 21)/NCB (n = 6) definition used in this study, we found CB tumors had higher immune scores (Wilcoxon p = 0.03; Fig. 3c) which positively correlated with PFS (Spearman ρ = 0.45, p = 0.017; Fig. 3d) in response to pembrolizumab + bevacizumab + metronomic cyclophophamide, supporting the predictive potential of our immune signature across the datasets. In contrast, metabolism scores showed no significant correlation with clinical benefit or PFS in this external cohort (Fig. 3e–f), consistent with our findings on inhouse data.

### Genomic profiling reveals no association between somatic alterations and treatment response

We next asked whether genomic alterations at baseline were associated with treatment outcomes. Whole-exome sequencing (WES) of pretreatment tumor biopsies revealed *TP53* mutations in the majority of cases in both D + O + C and D + C arms (Extended Data Fig. 1a-b). We examined DNA repair-related pathways and tumor mutational burden (TMB) because homologous recombination deficiency (HRD) is linked to elevated immune activity^31^, and higher tumor mutational burden (TMB) is also associated with improved survival and increased CD8^+^ T-cell infiltration in ovarian cancer^32^. In D + O + C, a pathogenic *ATM* missense mutation was identified in one exceptional responder, while another CB patient carried a pathogenic *MSH2* nonsense mutation (Extended Data Fig. 1, left). In D + C, an exceptional responder had multiple mutations in DNA repair genes (Extended Data Fig. 1, right). Pathogenic *PIK3CA* missense mutations were detected in two NCB patients in D + O + C and one in D + C (Extended Data Fig. 1). TMB was uniformly low across the treatment arms (Extended Data Fig. 1), suggesting that baseline genomic alterations are unlikely the major determinants of treatment benefit.

### Paired transcriptomic analysis reveals treatment-induced immune activation in patients with clinical benefit

**Figure 4:**
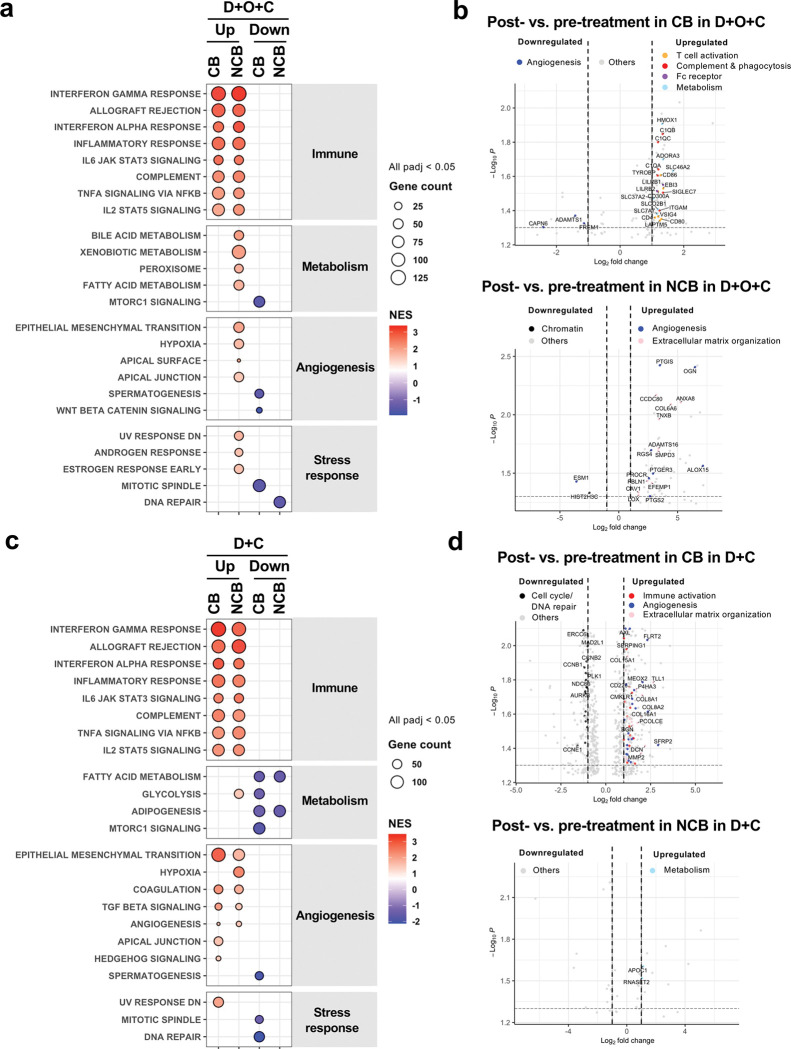
Paired transcriptomic analysis exhibits treatment-induced immune activation in patients with clinical benefit. **a.** Gene set enrichment analysis of bulk RNAseq data show the enrichment of upregulated or downregulated pathways after D+O+C treatment in tumors with CB or NCB. Bubble size = number of core genes contributing to the pathways. **b.** Bulk RNAseq data demonstrate the upregulated or downregulated genes after D+O+C treatment in tumors with CB (top) or NCB (bottom). **c.** Gene set enrichment analysis of bulk RNAseq data show the enrichment of upregulated or downregulated pathways after D+C treatment in tumors with CB or NCB. Bubble size = number of core genes contributing to the pathways. **d.** Bulk RNAseq data indicate the upregulated or downregulated genes after D+C treatment in tumors with CB (top) or NCB (bottom). Abbreviations: padj, adjusted p value; CB, clinical benefit; D+O+C, durvalumab, cediranib, and olaparib; D+C, durvalumab plus cediranib; NCB, no clinical benefit; NES, normalized enrichment score; PFS, progression-free survival.

Given the distinct baseline transcriptional patterns, we next assessed whether treatment induced dynamic shifts by analyzing paired pre- and on-treatment RNAseq from D + O + C (n = 11) and D + C (n = 9) patients. In D + O + C, CB tumors (n = 9) exhibited a macrophage-driven immune response (Fig. 4a, Supplementary Table 12), with upregulation of complement components, Fc receptors, and phagocytosis-related transcripts relative to baseline (Fig. 4b, Supplementary Table 13). This was accompanied by activation of interferon signaling and immunoregulatory pathways, together with elevated T cell markers and metabolic regulators, reflecting broad immune-metabolic remodeling toward an immune-dominant state (Fig. 4a–b, Supplementary Tables 12–13). Conversely, NCB tumors (n = 2) shifted toward developmental and stromal programs, enriched for angiogenesis and extracellular matrix (ECM) pathways (Fig. 4a, Supplementary Table 14). Concomitant downregulation of chromatin and endothelial transcripts suggested immune exclusion with enhanced tissue plasticity (Fig. 4b, Supplementary Table 14).

In D + C, CB tumors (n = 6) showed concurrent upregulation of immune and stromal remodeling pathways alongside downregulation of both mitotic spindle and DNA repair programs, indicating immune engagement coupled with suppressed proliferation (Fig. 4c–d, Supplementary Tables 15–16). In contrast, NCB tumors (n = 3) displayed increased glycolysis and reduced fatty acid metabolism and adipogenesis, consistent with their baseline metabolism-dependent phenotype (Fig. 4c–d, Supplementary Tables 15–16). Notably, NCB tumors exhibited only minimal gene-level changes (Fig. 4d, Supplementary Table 17), requiring further validation in large prospective studies.

### Peripheral immune cells and circulating tumor cells (CTCs)

**Figure 5:**
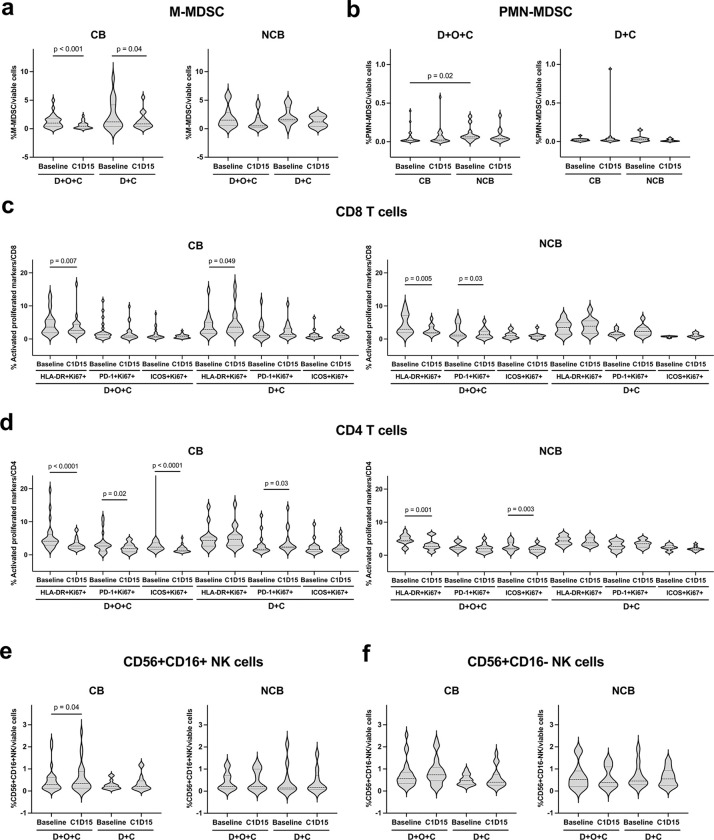
Circulating immune cell dynamics on treatment. **a.** Violin plots show the percentage of M-MDSCs among viable cells at baseline and C1D15 for patients with CB compared with NCB. **b.** Violin plots of the percentage of PMN-MDSCs among viable cells at baseline and C1D15 in both D+O+C and D+C arms. **c.** Frequencies of activated, proliferating CD8^+^ T cells (co-expressing Ki67 with HLA-DR, PD-1, or ICOS) at baseline and C1D15 in each arm for CB and NCB groups. **d.** Frequencies of activated, proliferating CD4^+^ T cells (co-expressing Ki67 with HLA-DR, PD-1, or ICOS) at baseline and C1D15 in each arm for CB and NCB groups. **e.** Violin plots indicate the percentage of CD56+ CD16+ NK cells among viable cells at baseline and C1D15 for patients with CB compared with NCB. **f.** Violin plots show the percentage of CD56+ CD16− NK cells among viable cells at baseline and C1D15 for patients with CB compared with NCB. Abbreviations: C1D15, cycle 1 day 15; CB, clinical benefit; D+O+C, durvalumab, cediranib, and olaparib; D+C, durvalumab plus cediranib; HLA-DR, human leukocyte antigen DR antigen; ICOS, inducible T-cell costimulatory; M-MDSC, monocytic myeloid-derived suppressor cells; NCB, no clinical benefit; NES, normalized enrichment score; PD-1, programmed cell death protein 1; PMN-MDSC, polymorphonuclear myeloid-derived suppressor cells; NK, natural killer.

We next evaluated the dynamic changes of circulating immune cells and CTCs following treatment with D + O + C (n = 36) or D + C (n = 26). Monocytic myeloid-derived suppressor cells (M-MDSC) significantly decreased by cycle 1 day 15 (C1D15) in the CB groups of both treatment arms (Wilcoxon p < 0.001 and 0.04, respectively; Fig. 5a), while no significant changes in NCB groups. Polymorphonuclear MDSC (PMN-MDSC) levels were comparable in pre- or post-treatment in CB and NCB groups for both D + O + C and D + C arms while PMN-MDCS levels were higher at baseline in the NCB group compared to that in the CB group of D + O + C arm (Mann–Whitney p = 0.02; Fig. 5b). Additionally, Fig. 5c–f show other immune cells including activated/proliferating T-cell subsets and NK cells. Lastly, CTC levels did not demonstrate significant changes over time and were not associated with PFS (Extended Data Fig. 2a-b).

### A cross-arm 18-gene NCB signature reflecting vascular remodeling and cytoskeletal plasticity predicts clinical outcome

**Figure 6:**
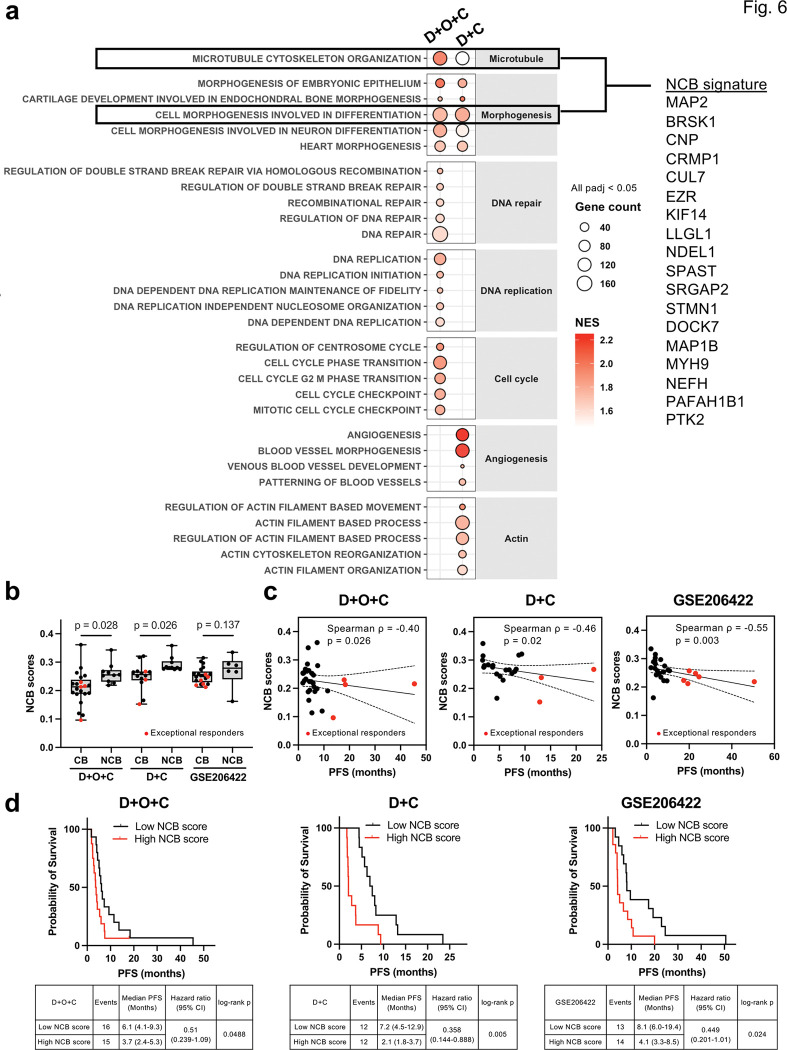
Baseline transcriptomic characteristics in patients with no clinical benefit. **a.** Gene set enrichment analysis of bulk RNAseq data indicate pathways that may have contributed to NCB (PD or SD < 4 months) or CB (PR or SD ≥ 4 months). Dot plot shows the enriched pathways associated with NCB. **b-d.** NCB signature scores in CB vs. NCB tumors within D+O+C, D+C and a public dataset (GSE206422; recurrent ovarian cancers treated with pembrolizumab, bevacizumab, and metronomic cyclophosphamide) calculated using singscore (b) and scores correlating with PFS (c-d). Two-sided Wilcoxon rank-sum test was used for comparing signature scores between CB and NCB. Spearman correlation test was used for correlation between signatures and PFS. Abbreviations: CB, clinical benefit; D+O+C, durvalumab, cediranib, and olaparib; D+C, durvalumab plus cediranib; NCB, no clinical benefit; PFS, progression-free survival.

While transcriptomic signatures of tumors with CB or exceptional response varied among the treatment arms, specifically, myeloid and interferon-mediated immune activation for D + O + C and concurrent enrichment of immune and metabolic pathways for D + C, tumors with NCB showed more consistent transcriptomic patterns across D + O + C and D + C arms. In NCB tumors, GSEA revealed upregulation of developmental morphogenesis and vascular adaptation signatures (all padj < 0.05; Fig. 6a and Supplementary Tables 2–3). These included VEGF-driven angiogenic processes alongside non-VEGF morphogenic programs, including WNT/β-catenin, TGF-β, Notch, and Hedgehog signaling and epithelial-mesenchymal transition (padj < 0.0001). We therefore hypothesized that NCB tumors may have bypassed VEGF blockade through alternate vascularization programs *e.g.*, developmental reprogramming^33, 34^, hence became less susceptible to D + C-based therapies.

NCB tumors also demonstrated upregulation of cytoskeletal organization and microtubule dynamics pathways (Fig. 6a and Supplementary Tables 2–3), indicating enhanced motility and cellular plasticity^35^. Additionally, in the D + O + C arm, NCB tumors were enriched for DNA repair, cell cycle checkpoint, and replication stress response pathways (all padj < 0.05, Fig. 6a and Supplementary Table 2), while D + C was not, potentially reflecting compensatory mechanisms in response to PARPi-induced genotoxic stress. In the D + C arm, NCB tumors upregulated angiogenesis and actin dynamics pathways upon treatment, while D + O + C did not.

To derive a compact resistance biomarker, we intersected the leading-edge genes from cell morphogenesis involved in differentiation and microtubule cytoskeleton organization pathways recurrently enriched in NCB tumors across both D + O + C and D + C arms (Fig. 6a and Supplementary Table 18). This yielded an 18-gene NCB signature (Supplementary Table 19). NCB scores were significantly higher in NCB than CB tumors within each arm (all p < 0.05; Fig. 6b), and a similar trend was observed in an external dataset treated with pembrolizumab, bevacizumab, and metronomic cyclophosphamide (GSE206422, Fig. 6b). Higher NCB scores correlated with shorter PFS in both our cohort and the external dataset (ρ ≤ − 0.4, p < 0.05; Fig. 6c), and patients with high NCB scores consistently experienced worse outcomes (Fig. 6d).

### MAP2 emerges as a recurrent NCB-associated gene and a potential biomarker of treatment resistance

**Figure 7:**
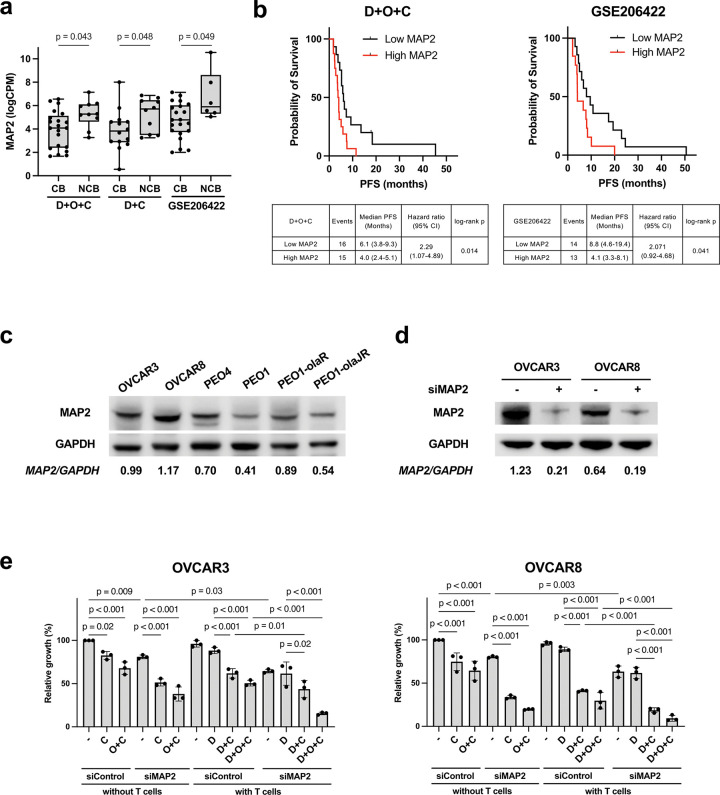
Silencing MAP2 enhances the efficacy of immunotherapy plus VEGFR and PARP inhibition. **a.** *MAP2* mRNA expression from RNAseq data of CB and NCB tumors in D+O+C, D+C, and GSE206422 datasets. LogCPM values of *MAP2* are shown. **b.** Kaplan-Meier analysis revealed that high *MAP2*expression was associated with significantly shorter PFS in D+O+C (left) and in GSE206422 dataset (right). **c.** Immunoblotting of baseline MAP2 protein levels was performed in a panel of ovarian cancer cell lines. **d.** MAP2 knockdown efficiency in platinum-resistant ovarian cancer cell lines (OVCAR3 and OVCAR8) was confirmed by immunoblotting. **e.** MAP2 knockdown using siRNAs against *MAP2* reduced T cell-mediated killing ability in platinum-resistant ovarian cancer cells (OVCAR3 and OVCAR8). Abbreviations: CB, clinical benefit; D+O+C, durvalumab, cediranib, and olaparib; D+C, durvalumab plus cediranib; LogCPM, log counts per million; MAP2, microtubule-associated protein 2; NCB, no clinical benefit; PFS, progression-free survival.

Cross-referencing the 18-gene signature with GSE206422 identified MAP2 as the only gene consistently upregulated in NCB tumors across both our cohort and the external dataset (p < 0.05; GSE206422, Fig. 7a). High MAP2 expression was significantly associated with inferior PFS in our D + O + C arm (median PFS 4.0 vs. 6.1 months; log-rank p = 0.01) and in GSE206422 (median PFS 4.1 vs. 8.8 months; log-rank p = 0.04; Fig. 7b). MAP2 encodes a cytoskeletal protein that stabilizes microtubules and regulates neuronal morphogenesis thus modulates microtubule dynamics^36^. Clinically, high MAP2 expression is associated with tumor invasion and lymph node metastasis in gastric cancer^37^, and is overexpressed in oral squamous cell carcinoma^38^. In preclinical studies, MAP2 upregulation increases oral cancer cell motility^38^ and confers resistance to microtubule-targeting agents in glioma cells^39^. Given its upregulation in NCB tumors across inhouse and public dataset (Fig. 7b), we hypothesized that MAP2 might promote cytoskeletal remodeling, enabling tumor cell plasticity and evasion of VEGF and PARP inhibition.

To functionally evaluate MAP2, we performed MAP2 siRNA knockdown in MAP2-high expressing, platinum-resistant ovarian cancer cell lines (OVCAR3 and OVCAR8). After MAP2 knockdown, cells were treated with cediranib (10 μM), durvalumab (10 μg/mL), and/or olaparib (10 μM) (Fig. 7c). MAP2 knockdown modestly altered CD8^+^ T-cell–mediated killing under D + O + C or D + C treatment compared to control conditions (Fig. 7d–e), suggesting a potential role of MAP2 in shaping tumor susceptibility to immune cytotoxicity. Taken together, although exploratory, these data suggest MAP2 may partly contribute to an immune-excluded phenotype in NCB tumors.

## Discussion

Platinum-resistant EOC remains a high-mortality setting with few treatment options^1, 2^. Recent experience highlights that rational biomarker selection is crucial for success. For example, MIRV didn’t show clinical efficacy in an unselected population but demonstrated clear benefit in FRα-high tumors, leading to a biomarker-defined SOC^40^. By analogy, strategies combining DNA damage response inhibitors, anti-angiogenic agents, and PD-(L)1 blockade will likely require prospective biomarker-driven selection. Although preclinical studies suggested synergy among PARP, VEGF, and ICI combinations^22,23,25^, the randomized NRG-GY023 trial showed limited benefit of olaparib plus cediranib with immunotherapy in bevacizumab-pretreated patients (8.3 months vs. 7.5 months for SOC)^41^. Our proof-of-concept study addressed this gap by identifying biological determinants of response and resistance to D + O + C and D + C.

Baseline transcriptomic profiling revealed two distinct axes of benefit. Across both arms, immune-active tumors were associated with exceptional responders, consistent with prior studies showing that HRD and interferon-primed CD8^+^ T cells predict PARPi + ICI sensitivity^42^. Additionally, comprehensive immunogenomic analyses in HGSOC have further shown that BRCAness and high immune infiltration correlate with increased vulnerability to combination immunotherapy, and that genomic instability activates innate immune pathways, enhancing response to PARPi and ICI combination^43^. In the D + C arm, exceptional responders also tracked with metabolic enrichment, suggesting that when PARP inhibition is absent, efficacy depends on immunometabolic fitness. This is biologically plausible, as T-cell effector function relies on oxidative and cholesterol-linked metabolism^44^, while VEGF blockade enhances vascular normalization and chemokine-mediated infiltration but requires metabolically fit CD8^+^ T cells to sustain tumor entry^45, 46^. Collectively, these findings support that D + O + C efficacy is primarily immune-driven, amplified by PARP-induced interferon signaling, whereas D + C efficacy requires complementary metabolic activation.

In contrast, tumors from NCB patients displayed convergent enrichment of angiogenic escape and cytoskeletal remodeling, indicating a multifaceted resistance program. VEGF-dependent and alternative developmental signals (e.g., Wnt/β-catenin, TGF-β, Notch, Hedgehog) were upregulated, consistent with mechanisms enabling escape from VEGF/VEGFR blockade^34, 47^. Prior reports link anti-VEGF resistance in ovarian cancer to alternative angiogenic pathways and to immunosuppressive microenvironments fostered by hypoxia-driven MDSC recruitment^33^. Furthermore, enrichment of cytoskeletal and microtubule remodeling signatures including epithelial-mesenchymal transition suggests invasive, mesenchymal-like states that coincide with therapy resistance^35^. Together, these pathways likely enforce immune exclusion and resilience under combined VEGF, PD-(L)1, and PARP blockade.

Exploratory analyses identified an 18-gene NCB signature enriched across arms and associated with inferior PFS in both our cohort and an independent ICI + VEGF dataset^30^. Within this set, MAP2 was the only gene consistently upregulated in NCB tumors across datasets and was associated with shorter PFS. MAP2 encodes a microtubule-stabilizing, neurofilament-associated protein^48^, previously linked to invasion, motility, and resistance to microtubule-targeting agents in oral cancer and glioma cells^38, 39^. Its elevated expression in NCB tumors may reflect activation of neurogenic transcriptional circuits, consistent with emerging evidence that cancer-nerve interactions^49, 50^ and neuro-immune crosstalk^51^ can foster immune checkpoint resistance. Although exploratory, these findings raise the hypothesis that targeting MAP2-related pathways may enhance efficacy of immunotherapy plus anti-VEGF regimens. In this context, the phase III ENGOT-ov65 trial (NCT05116189) is evaluating pembrolizumab plus weekly paclitaxel with or without bevacizumab^52^. Early reports indicate a significant OS benefit in PD-L1–positive tumors compared to chemotherapy ± bevacizumab^53^. The addition of an anti-microtubule agent to ICI + anti-VEGF therapy aligns with our hypothesis that cytoskeletal programs contribute to resistance, and suggests that integrating taxanes or other microtubule-targeting strategies could counteract this axis. Importantly, OS benefit was confined to the PD-L1–high subgroup, suggesting the interplay between baseline immune phenotypes and cytoskeletal vulnerability. Nevertheless, these links remain speculative, and dedicated mechanistic studies will be needed to establish whether MAP2 or related cytoskeletal factors are actionable determinants of response in recurrent EOC.

The limitation of our study includes the nature of a single-center, non-randomized clinical trial without a direct comparator arm. Also, the modest sample size may limit overall statistical power, particularly for subgroup analyses such as those involving exceptional responders. As such, our findings should be interpreted with caution and as hypothesis-generating. Additional limitations include the exploratory nature of the biomarker analyses and the limited number of paired transcriptomic samples, which may affect generalizability. The mechanistic insights regarding MAP2 are based on *in vitro* knockdown models and will require validation *in vivo* and in larger, independent cohorts. Finally, bulk RNAseq cannot capture spatial immune architecture or cellular heterogeneity; future studies incorporating single-cell and spatial profiling will be important to fully delineate the immune-metabolic-structural interactions influencing therapeutic outcomes.

Taken together, our data support assignable axes of benefit in recurrent EOC: an immune-primed axis (most relevant to PARPi-containing backbones) and an immunometabolic axis (relevant to anti-VEGF + ICI). Resistance converges on alternative vascularization and cytoskeletal plasticity, for which our NCB signature/MAP2 provide negative predictors and therapeutic hypotheses. Future studies may employ biomarker-stratified randomization (immune-high vs metabolic-high vs NCB signature-high), embed serial tissue/blood profiling to verify on-target remodeling, and pre-specify adaptive add-ons for NCB biology. This strategy offers a credible path to convert short-lived control into durable benefit for defined subgroups of patients with platinum-resistant recurrent EOC.

## Methods

Detailed descriptions of the methods, including WES, RNAseq, and CTC analyses, are provided in the Supplementary Materials.

### Clinical trial

#### Study design and participants

This study describes the phase II ovarian cancer cohort (cohort 1) of an open-label, multi-cohort, multi-arm, single-center phase I/II study (NCT02484404)^23, 54, 55^. Patients enrolled were ≥ 18 years of age with histologically or cytologically confirmed recurrent ovarian, fallopian tube, or primary peritoneal cancer that were willing and able to undergo fresh pre-treatment core biopsies. Other eligibility criteria included measurable disease by RECIST v1.1, Eastern Cooperative Oncology Group (ECOG) status ≤ 2 and adequate organ function. Patients previously treated with ICI except durvalumab or treated with olaparib or bevacizumab were eligible. Patient were assigned to the D + O + C arm, or D + C arm, or D + O arm, as per investigator’s discretion based on prior treatment history. The D + O + C arm enrolled patients with high-grade serous or clear-cell histology and D + C arm enrolled those with all histologies. All participants provided written informed consent before enrollment and on using clinical samples for research. The study has been conducted in accordance with ethical principles that have their origin in the Declaration of Helsinki and are consistent with the International Council on Harmonization guidelines on Good Clinical Practice, all applicable laws and regulatory requirements, and all conditions required by a regulatory authority and/or institutional review board. The study protocol was approved by the Institutional Review Board of the Center for Cancer Research, National Cancer Institute.

#### Clinical trial procedures

Patients in the D + O + C and D + C arms received durvalumab 1,500 mg intravenously every 4 weeks, in combination with cediranib 20 mg orally once daily, administered on a 5-days-on/2-days-off schedule with or without olaparib 300 mg orally twice daily. Treatment was given in 28-day cycles and continued until disease progression, unacceptable toxicity, or withdrawal of consent. Serial blood samples were collected at baseline, C1D15, cycle 3 day 1 (C3D1), and at disease progression. Mandatory fresh core biopsies were obtained at baseline (within 24–48 hours prior to C1D1), and optional on-treatment tumor biopsies prior to C1D15, C3D1 and at progression. Radiologic assessments were conducted at baseline and every 2 cycles (+/− 1 week) using CT or MRI. Tumor responses were evaluated according to investigator-assessed RECIST v1.1 criteria. Patients were considered RECIST-evaluable for treatment response if they completed at least one post-treatment imaging assessment. Adverse events (AEs) were recorded at each study visit and graded per Common Terminology Criteria for Adverse Events version 4.0 (CTCAE v4.0). Safety analyses included all patients who received at least one dose of study treatment.

#### Study objectives and endpoints

The primary objective was objective response rate (ORR; defined as the proportion of patients in each group with complete (CR) or confirmed/unconfirmed PR per investigator-assessed RECIST v1.1.

Secondary objectives included PFS, safety and tolerability according to the NCI CTCAE v4.0. PFS was defined as the time from enrollment to the first documentation of disease progression or death.

#### Single-sample gene set scoring

In D + O + C, 20 genes annotated to the GO term positive regulation of immune response were selected as the immune signature, while in D + C, 15 genes annotated to sterol metabolic process were used as the metabolic signature. Shared pathways enriched in NCB tumors across both treatment arms were identified by intersecting significant gene sets. Leading-edge subsets from each arm were extracted for the overlapping pathways in cell morphogenesis and microtubule cytoskeleton organization. Intersection of leading-edge genes across arms yielded an 18-gene NCB core signature. We quantified the relative activity of immune and metabolic gene signatures using the singscore^27^ R/Bioconductor package (version 1.29.0). Resulting signature scores were merged with clinical metadata, including clinical benefit status and PFS.

In the external dataset (GSE206422), raw read counts were downloaded from the Gene Expression Omnibus (GEO) and normalized using the variance-stabilizing transformation in DESeq2. Clinical data were extracted from the associated publication, including PFS and best response per RECIST. Variance-stabilized expression values for each signature gene were z-scored across samples and averaged to compute a per-sample immune signature, metabolism signature, and NCB signature scores. Samples were stratified by CB and NCB using the same thresholds as in our in-house analysis.

#### Immune cell subset analysis

For Immune cell subset analysis, peripheral blood specimens were collected in cell preparation tubes with sodium citrate at baseline (pre-treatment), prior to C1D15, C3D1, and at disease progression. PBMCs were obtained isolated from whole blood by centrifugation and viably frozen until analysis. On the day of analysis, frozen PBMCs were thawed and washed with PBS, followed by incubation with an Fc receptor blocking reagent (#130–059-901, Miltenyi Biotec, Gaithersburg, MD, USA) and stained with monoclonal antibodies for 20 minutes at 4°C. Dead cells were excluded from the analysis using LIVE/DEAD Fixable Aqua viability dye. All analyses were performed using multiparametric flow cytometry (MACSQuant; Miltenyi Biotec). Data were analyzed using FlowJo software v.10.6.1 (FlowJo, LLC, OR, USA). Comprehensive immune flow analysis involved enumeration of immune subsets including activated CD4 and CD8 subsets, regulatory T-cells, memory T-cells, MDSCs, monocytes dendritic cells and NK cells along with their activation markers.

### In vitro study

#### Ovarian cancer cell lines

PEO1 (*BRCA2* mutation 5193C > G, #10032308-1VL) and PEO4 (*BRCA2* reversion mutation, #10032309-1VL) were purchased from MilliporeSigma (Rockville, MD, USA). OVCAR3 and OVCAR8 (platinum-resistant *BRCA* wild-type HGSOC) were received from NCI-60 collection at the NCI Frederick (Frederick, MD, USA). PARPi-resistant derivatives included PEO1-olaR (gift from Dr. Benjamin Bitler, University of Colorado) and PEO1-olaJR^56^, generated in-house as previously described. All cell lines were cultured in RPMI1640 with medium L-glutamine (#11875119, Life Technologies, Frederick, MD, USA) and supplemented with 10% fetal bovine serum (FBS), 1% penicillin/streptomycin, 1 mM sodium pyruvate and 5μg/ml of insulin from bovine pancreas (#I0516, MilliporeSigma). PEO1-olaR was routinely maintained at 2 μM of olaparib while PEO1-olaJR at 20 μM of olaparib. Cells were cultured without olaparib for at least 3 days prior to experiments. All cell lines were routinely tested for *Mycoplasma* using MycoAlert Mycoplasma Detection Kit (#LT-07-318, Lonza, Portsmouth, NH, USA).

#### CD8^+^ T Cell isolation and activation

Primary human CD8^+^ T cells (#PCS-800-017, ATCC, Manassas, VA, USA) were thawed and cultured in RPMI-1640 supplemented with 10% FBS and recombinant human IL-2 (50 IU/mL, 200-02-50UG, ThermoFisher Scientific, Rockville, MD, USA). Cell density was adjusted to 1 × 10^6^ cells/mL, and the culture medium was refreshed every 2 days. On day 10, the CD8 + T cells were restimulated with CD3/CD28 Dynabeads (#11161D, Thermo Fisher Scientific) at a 1:1 bead-to-cell ratio and incubated at 37°C, 5% CO_2_. On day 15 post-activation, the cells were used for co-culture assays.

#### siRNA transfection

ON-TARGETplus SMARTpool-Human of *MAP2* (#L-007299-00-0005) siRNAs and Dharmafect 1 reagent (#T-2001-02) were used for gene knockdown experiments as per manufacturer’s protocol (Horizon Discovery, Lafayette, CO, USA). Non-targeting control siRNAs (#D-001810-10-20, Horizon Discovery) were used as negative control. Cells transfected with siRNA targeting *MAP2* were seeded at 5×10^4^ cells per well in 24-well plates for trypan blue cell counting. Knockdown efficiency was confirmed by immunoblotting 48 hours post-transfection.

#### Immunoblotting

Cells were collected for protein extraction and subjected to immunoblotting. Blots were visualized using the Licor Odyssey Imaging System. MAP2 (#4542), ECL goat anti-rabbit IgG HRP (#7074) and GAPDH (#5174) antibodies were purchased from Cell Signaling Technology (Danvers, MA, USA).

#### Drug preparation

For *in vitro* assays, PARPi olaparib (#S1060) was purchased from Selleck Chemicals (Houston, TX, USA). Durvalumab (#HY-P9919), cediranib (#HY-10205) were from MedCahemExpress (Monmouth Junction, NJ, USA). 100 mM of olaparib as well as 10 mM of cediranib were prepared as stocks in dimethyl sulfoxide (DMSO; #S-002-M, MilliporeSigma) and stored in aliquots at −80°C until use.

#### Cell growth assay

Cells transfected with siRNA targeting *MAP2* were seeded at 5 × 10^4^ cells per well in 24-well plates and pretreated for 24 hours with cediranib (10 μM), olaparib (10 μM), durvalumab (10 μg/mL), or their combinations. Following drug pretreatment, activated CD8^+^ T cells were added at an effector-to-target (E:T) ratio of 3:1 and co-cultured for 48 hours in drug-containing medium without IL-2. After co-culture, non-adherent CD8^+^ T cells were gently removed by washing twice with PBS and collected for viability assessment using trypan blue staining and cell counting.

### Statistical analyses

Each arm’s design followed a Simon optimal two-stage approach^57^. The D + O + C arm was designed to test an ORR improvement from 20% to 40% (p0 = 0.20, p1 = 0.40) with α = 0.10 and β = 0.10. In the first stage, 17 patients were to be enrolled. If ≥ 4 responses in the first 17 patients, the second stage would enroll additional 20 patients, with 11 or more responders of 37 patients (29.7%) would be considered positive for further development. Similarly, the D + C arm, the design aimed to rule out a ORR of 10% in favor of a target ORR of 30%, with a one-sided α = 0.10 and β = 0.10. In the first stage, 12 patients were to be enrolled. If 2 or more responses occurred in the first 12 patients, accrual would continue to a total of 35 patients. 6 or more responses out of 35 (17.1%) would be considered positive. Safety analyses included all patients. Unfortunately, the trial was stopped early due to COVID-19, slow accrual and drug supply issues, therefore approximately 90% of planned enrollment was achieved.

ORR, CB, and associated 95% CIs were calculated using the Clopper–Pearson method. Median PFS was estimated using Kaplan–Meier method; patients without progression were censored at last follow-up on May 13, 2025.

Statistical equivalence testing was performed using the TOSTER package developed for the R statistical-computing software (version 4.5.1; R Foundation for Statistical Computing, Vienna, Austria). For equivalence analysis of clinical outcomes, censored PFS values (constituting only ~ 3% of the observations) were excluded, as standard equivalence tests are not suitable for censored data. The equivalence margin for PFS was set to 1.5 months. Sample normality for PFS was assessed using the Shapiro–Wilk test and rejected (p < 0.05). Consequently, statistical equivalence between the D + O + C and D + C arms in both ITT and evaluable patients was evaluated for PFS using a nonparametric Wilcoxon-based equivalence test. For the ORR, DCR, and CB values in both populations, we used an equivalence test developed specifically for proportions. The equivalence margin for these values was set to 0.15. As equivalence between the D + O + C and D + C arms could not be demonstrated (p > 0.05) for any of the comparisons, exploratory biomarker analyses were conducted separately to avoid confounding treatment-specific effects.

Group means of immune and metabolic signature scores were compared using the Wilcoxon signed rank-sum test. Spearman’s rank correlation was used to assess the association between immune and metabolism signature scores across patients. Also, dynamic changes of immune cells between baseline and on-treatment were compared using the Wilcoxon matched-pairs signed rank test. Mann-Whitney test was used between treatment arms at the same sampling time point.

For *in vitro* studies, all experiments were performed in triplicate. Data were analyzed using one-way AMOVA for multiple comparison and are shown as mean ± standard deviation (SD). The p < 0.05 were considered significant. All statistical analyses were done using GraphPad Prism v10.

## Supplementary Material

Supplementary information

Extended Data Figures 1–2. Supplementary Tables 1–19.

Supplementary Files

This is a list of supplementary fi les associated with this preprint. Click to download.

• ExtendedData12.docx

• ClinicalProtocol20210909.docx

• FinalSupplmentaryTables.xlsx

• nrreportingsummary.pdf

## Figures and Tables

**Table 1:** Baseline Characteristics

	D + O + C (n = 39)	D + C (n = 29)
Age in years, median (range)	64 (33–79)	63 (46–78)
ECOG performance status at start of trial, N (%)		
0	11 (28.2%)	12 (41.4%)
1	24 (61.5%)	15 (51.7%)
2	4 (10.3%)	2 (6.9%)
Primary site, N (%)		
Ovary/fallopian tube	39 (100.0%)	27 (93.1%)
Primary peritoneum	0	2 (6.9%)
Histology, N (%)		
High grade serous	34 (87.2%)	21 (72.4%)
Clear cell	5 (12.8%)	2 (6.9%)
Granulosa cell	0	3 (10.3%)
Carcinosarcoma	0	2 (6.9%)
High grade endometrioid	0	1 (3.4%)
BRCA mutation status, N (%)		
Germline	4 (10.3%)	3 (10.3%)
Somatic	2 (5.1%)	0
Wild-type	33 (84.6%)	25 (86.2%)
Unknown	0	1 (3.4%)
Race, N (%)		
White	26 (66.7%)	19 (65.5%)
Black or African American	7 (17.9%)	2 (6.9%)
Asian	5 (12.8%)	6 (20.7%)
Unknown	1 (2.6%)	2 (6.9%)
Platinum sensitivity, N (%)		
Sensitive	6 (15.4%)	9 (31.0%)
Resistant, primary	11 (28.2%)	4 (13.8%)
Resistant, secondary	18 (46.2%)	14 (48.3%)
Refractory	4 (10.3%)	2 (6.9%)
Lines of prior systemic therapy, N (%)		
1	1 (2.6%)	1 (3.4%)
2–3	24 (61.5%)	15 (51.7%)
≥ 4	14 (35.9%)	13 (44.8%)
Lines of prior cytotoxic therapy		
1	3 (7.7%)	4 (13.8%)
2–3	29 (74.4%)	16 (55.2%)
≥ 4	7 (17.9%)	9 (31.0%)
PARP inhibitor	15 (38.5%)	19 (65.0%)
Bevacizumab	31 (79.5%)	17 (58.6%)
Anti PD-1 antibody	4 (10.3%)	0
Vaccine or other immunotherapy	3 (7.7%)	5 (17.2%)
Hormonal therapy	8 (20.5%)	6 (20.7%)

Notes: Patients were defined as having primary platinum-resistant disease if relapse of disease occurred < 6 months after completing first-line platinum-based therapy, having secondary platinum-resistant disease if progression of disease occurred ≥ 6 months after first-line platinum-based therapy but < 6 months after second-line or last line platinum-based therapy, and having primary platinum-refractory disease if progression of disease occurred during first-line platinum-based therapy.

Abbreviations: ECOG, Eastern Cooperative Oncology Group; PARP, poly (ADP-ribose) polymerase; PD-1,programmed cell death protein 1.

## Data Availability

All data supporting the findings of this study are available within the article and/or its supplementary materials. The study protocol is available in the Supplementary Information.
